# Association of admission blood glucose level and clinical outcomes in elderly community‐acquired pneumonia patients with or without diabetes

**DOI:** 10.1111/crj.13526

**Published:** 2022-07-24

**Authors:** Weijian Zeng, Xiaoxing Huang, Weijie Luo, Mingqian Chen

**Affiliations:** ^1^ Department of Critical Care Medicine Meizhou People's Hospital (Huangtang Hospital) Meizhou China

**Keywords:** admission blood glucose, clinical outcomes, community‐acquired pneumonia, diabetes mellitus, elderly patients

## Abstract

**Introduction:**

Community‐acquired pneumonia (CAP) is the major cause of infection‐related mortality worldwide. Patients with CAP frequently present with admission hyperglycemia.

**Objectives:**

The aim of this study was to evaluate the association between admission blood glucose (ABG) level and clinical outcomes in elderly CAP patients (≥80 years of age) with or without diabetes.

**Methods:**

In this single center retrospective study, 290 elderly patients diagnosed with CAP were included. Demographic and clinical information were collected and compared. The associations between admission blood glucose level and the 30‐day mortality as well as intensive care unit (ICU) admission and invasive mechanical ventilation (IMV) in elderly CAP patients with or without diabetes were assessed.

**Results:**

Of the 290 eligible patients with CAP, 159 (66.5%) patients were male, and 64 (22.1%) had a known history of diabetes at hospital admission. After adjusting for age and sex, the logistic regression analysis had identified several risk factors that might be associated with clinical outcomes in elderly patients with CAP. Multivariable logistic regression analysis revealed that admission glucose level > 11.1 mmol/L was significant associated with ICU admission, IMV, and 30‐day mortality both in non‐diabetic and diabetic patients. Furthermore, Kaplan–Meier analysis indicated that patients with higher admission glucose level were correlated statistically significantly with 30‐day mortality in patients with CAP (*P* < 0.001).

**Conclusion:**

Admission blood glucose is correlated with 30‐day hospital mortality, ICU admission, and IMV of CAP in elderly patients with and without diabetes. Specially, admission glucose > 11.1 mmol/L was a significant risk factor for 30‐day hospital mortality.

## Graphical abstract

This study aimed to investigate the association between admission blood glucose level and clinical outcomes, including 30‐day mortality, ICU admission, and invasive mechanical ventilation, in elderly CAP patients with or without diabetes. The results showed that higher admission blood glucose is correlated with 30‐day hospital mortality, ICU admission, and IMV of elderly CAP patients with and without diabetes. Specially, admission glucose > 11.1 mmol/L were correlated statistically significantly with 30‐day hospital mortality in elderly CAP patients.

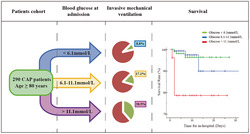

## 1 INTRODUCTION

Community‐acquired pneumonia (CAP) is a commonly encountered pulmonary infectious disease in clinical practice.^1^ It is related to diverse causes with complicated pathogen, and its severity varies dramatically from one person to another. CAP is responsible for the high rate of morbidity and mortality in adults from both developed and developing countries, with an estimated annual incidence ranging from 1.6 to 11 per 1000 adult population, which claims a considerable number of lives each year.^2^, ^3^ More seriously, the number of patients hospitalized with CAP has been increasing in China, especially among the elderly.^4^ Sheng et al. reported in a retrospective analysis of 27 723 hospitalized patients with pneumonia in Shanghai in 2011, which showed that older age groups have a higher incidence, accounting for 44.6% of the total.^5^ Another study from Hong Kong in 2016 reported that in 197 316 emergency hospital admissions due to pneumonia, patients older than 65 years as the most common population, accounting for 73.8% of cases.^6^ Indeed, it contributes to poor clinical outcomes and increased expenditure burden and medical resources for patients and society at large.

Nowadays, identifying risk factors of CAP prognosis and mortality may provide basis for timely and effective treatment of patients with CAP.^7^ Diabetes mellitus is a highly prevalent chronic metabolic disorder that occurs in approximately 5–10% in the older population.^8^ Moreover, with the increase in the prevalence of diabetes, the incidence of CAP also increases significantly. Interestingly, several studies have reported that hyperglycemia on admission is an independent risk factor for severe clinical outcomes among patients hospitalized with pneumonia or other respiratory diseases.^9^, ^10^, ^11^ Nonetheless, if admission glucose levels with different diabetes statuses could have a significant effects on elderly patients with CAP have not been well characterized. Therefore, the aim of present study was to evaluate the potential relationship between admission glucose level and the clinical outcomes in elderly patients with and without diabetes presenting with CAP in a hospital‐based retrospective cohort study.

## 2 MATERIALS AND METHODS

### 2.1 Study design and participants

**FIGURE 1:**
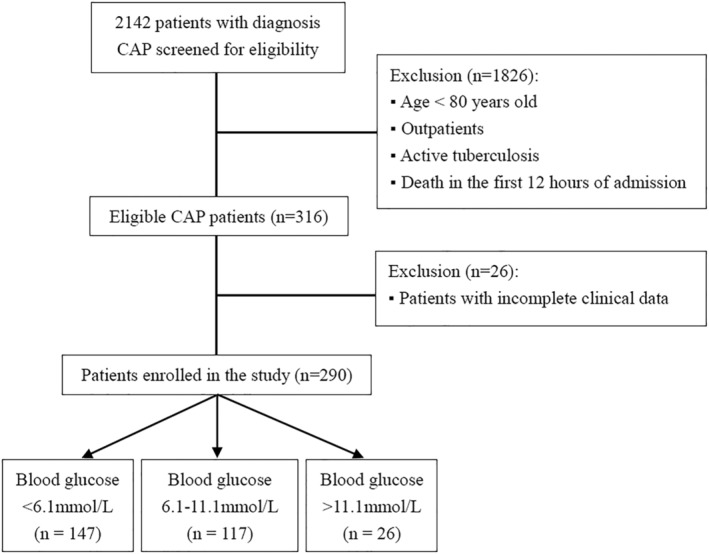
Flow chart of patient recruitment

The present study was a retrospective observational study conducted at Meizhou People's Hospital, which is a large tertiary hospital in the south of China with over 3000 inpatient beds. We observed 2142 consecutive hospitalized CAP patients that were admitted to Meizhou People's hospital from January 2016 to December 2020. The inclusion criterion was the patient discharged with a confirmed diagnosis of CAP according to the 2016 CAP clinical practice guidelines by the Chinese Thoracic Society.^12^ The patients were excluded based on the following criteria (Figure 1): (1) aged <80 years; (2) outpatients; (3) with active tuberculosis; (4) patients with incomplete data in medical records. Finally, 290 patients were included for analysis in this study. The study protocol was reviewed and approved by the Meizhou People's Hospital institutional ethical committee. All procedures complied with the ethical guidelines outlined in the 1975 Helsinki Declaration. Patient consent was waived due to the retrospective nature and observational study design.

### 2.2 Definitions

CAP was defined as the presence of acute illness with features of lower respiratory tract infection and the presence of consolidation in the chest CT or radiography radiograph. The patients were classified into diabetes and non‐diabetes groups according to known diabetes status on admission only. All patients that had admission blood glucose measured were also assigned to three groups in terms of admission glucose level (<6.1 mmol/L, 6.1–11.1 mmol/L, and >11.1 mmol/L) for analysis. Elderly patients were defined as those aged 80 years and above.

### 2.3 Data collection and clinical outcomes

Initial data after admission contained demographic, baseline clinical characteristics, and laboratory findings were extracted from the electronic medical record system. For the examination of admission plasma glucose, blood samples were collected within the within 3 h once their admission. The primary outcome was defined as 30‐day hospital mortality. The secondary outcomes were consisting of intensive care unit (ICU) admission and/or need for invasive mechanical ventilation (IMV). All patients included in this study were followed up for discharge status (survival/death) or death within 30 days.

### 2.4 Statistical analysis

Statistical Packages for Social Sciences (SPSS) software version 20.0. (IBM corp., Armonk, NY, USA) was used for the statistical analysis. Continuous variables are described as medians and interquartile ranges (IQRs). Two‐group comparisons were analyzed by Mann–Whitney *U* test, and Kruskal–Wallis test was used to compare more than two groups. Categorical variables were presented as numbers and percentages, and χ^2^ test or Fisher's exact test was utilized to compare their difference, as appropriate. Multivariate logistic analysis for clinical outcomes was performed. The model was adjusted for age and sex. Survival was estimated by the Kaplan–Meier method, and the differences in survival rates were evaluated with a log‐rank test. A *P* value of <0.01 was considered statistical significance.

## 3 RESULTS

### 3.1 Patient demographic and clinical characteristics

Out of 2142 patients who presented with CAP from January 2016 to December 2020, a total of 1826 patients including age <80 years old, outpatients, with active tuberculosis, and death in the first 12 h of admission were therefore excluded. Another, 26 patients without complete clinical data were also ruled out. Finally, 290 elderly patients with CAP were included in the analysis, as shown in Figure 1. The remaining patients consisted of 159 (66.5%) male and 131(33.5%) female aged 80–98 years.

The demographic and clinical characteristics of the study population are summarized in Table 1, among them, 64 (22.1%) patients with a prior diagnosis of diabetes and 226 (77.9%) of the 290 patients without a history of diabetes. The percentages of hypertension in CAP patients with diabetes were higher than those without diabetes (*P* < 0.001). However, there were no obvious differences in the incidences of IMV, ICU admission, hospital length of stay, and 30‐day mortality between patients with and without diabetes (all *P* > 0.01). Additionally, patients were divided into 3 groups according to the admission blood glucose level ranging from <6.1, 6.1–11.1, and >11.1 mmol/L, respectively. The percentages of chronic kidney disease and chronic liver disease, as well as the incidences of fatigue and impaired consciousness, and CURB‐65 score show differences among the three groups. Moreover, significant differences in the severe CAP on admission, IMV, ICU admission, and 30‐day mortality were also found among the three groups (*P* < 0.01).

**TABLE 1:** Patients' demographic and clinical characteristics admission to the hospital

	Diabetes (*n* = 64)	Non‐diabetes (*n* = 226)	*P* value	Blood glucose < 6.1 mmol/L (*n* = 147)	Blood glucose 6.1–11.1 mmol/L (*n* = 117)	Blood glucose > 11.1 mmol/L (*n* = 26)	*P* value
Demographics							
Age, years	85.1 ± 4.2	85.4 ± 4.1	0.693	85.0 ± 4.1	85.6 ± 4.1	85.6 ± 4.5	0.504
Male, *n* (%)	29 (45.3)	130 (57.5)	0.083	85 (57.8)	65 (55.6)	9 (34.6)	0.089
Smoking, *n* (%)	13 (20.3)	66 (29.2)	0.158	44 (29.9)	32 (27.4)	3 (11.5)	0.152
Alcohol intake, *n* (%)	13 (20.3)	45 (19.9)	0.944	33 (22.4)	24 (20.5)	1 (3.8)	0.090
Systolic blood pressure, mm Hg	137.4 ± 26.5	134.6 ± 21.8	0.390	134.7 ± 20.8	136.9 ± 22.4	130.6 ± 34.0	0.418
Diastolic blood pressure, mm Hg	78.4 ± 12.7	77.6 ± 12.9	0.658	77. 9 ± 11.5	78.8 ± 13.9	72.4 ± 17.8	0.068
Pre‐existing comorbidities, *n* (%)							
Hypertension	41 (64.1)	88 (38.9)	<0.001	59 (40.1)	55 (47.0)	15 (57.7)	0.196
Cardiovascular disease	24 (37.5)	55 (24.3)	0.037	35 (23.8)	35 (29.9)	9 (34.6)	0.336
Cardiac failure	4 (6.3)	4 (1.8)	0.053	2 (1.4)	4 (3.4)	2 (7.7)	0.213
Chronic obstructive pulmonary disease	0 (0)	11 (4.9)	0.153	5 (3.4)	6 (5.1)	0 (0)	0.274
Chronic kidney disease	14 (21.9)	51 (22.6)	0.907	24 (16.3)	30 (25.6)	11 (42.3)	0.008
Chronic liver disease	7 (10.9)	14 (6.2)	0.196	9 (6.1)	6 (5.1)	6 (23.1)	0.005
Symptoms at presentation, *n* (%)							
Fatigue	12 (18.8)	51 (22.6)	0.513	23 (15.6)	30 (25.6)	10 (38.5)	0.014
Impaired consciousness	12 (18.8)	39 (17.3)	0.782	14 (9.5)	25 (21.4)	12 (46.2)	<0.001
CURB‐65 score, *n* (%)							
1–2	46 (71.9)	172 (76.1)	0.478	122 (83.0)	85 (72.6)	11 (42.3)	<0.001
3–4	18 (28.1)	54 (23.9)		25 (17.0)	32 (27.4)	15 (57.7)	
Clinical outcomes							
Invasive mechanical ventilation	14 (21.9)	50 (22.1)	0.966	18 (12.2)	33 (28.2)	13 (50.0)	<0.001
ICU admission	17 (26.6)	88 (38.9)	0.069	60 (40.8)	30 (25.6)	15 (57.7)	0.002
Hospital length of stay (days)^a^	6.0 (4.0–8.0)	6.0 (4.0–8.0)	0.561	5.0 (4.0–8.0)	6.0 (4.0–8.0)	5.0 (2.0–12.5)	0.591
30‐day mortality (%)	3 (4.69)	8 (3.54)	0.957	3 (2.04)	3 (2.56)	5 (19.23)	0.004

*Note*: Values are expressed as the mean ± standard deviation or number (percentage).

Abbreviation: ICU, intensive care unit.

^a^ Data were expressed as medians (interquartile range).

### 3.2 Laboratory parameters

As shown in Table 2, laboratory parameters at admission were compared. CAP patients with diabetes had a higher white blood cell count, neutrophil count, higher level of uric acid, urea nitrogen, and triglycerides, compared with the non‐diabetes patients group (*P* < 0.01). Furthermore, patients with blood glucose levels 6.1–11.1 or >11.1 mmol/L had a trend towards higher white blood cell count, neutrophil count, higher level of uric acid, urea nitrogen, procalcitonin, C‐reactive protein (CRP), and D‐dimer compared with patients with blood glucose levels <6.1 mmol/L.

**TABLE 2:** Comparison of laboratory parameters among subgroups at admission

	Diabetes (*n* = 64)	Non‐diabetes (*n* = 226)	*P* value	Blood glucose < 6.1 mmol/L (*n* = 147)	Blood glucose 6.1–11.1 mmol/L (*n* = 117)	Blood glucose > 11.1 mmol/L (*n* = 26)	*P* value
White blood cell count, ×10^9^/L	11.6(8.43–15.8)	9.10(6.60–12.8)	0.001	8.20(6.30–12.0)	10.6(8.20–14.6)	12.1(9.83–14.8)	<0.001
Neutrophil count, ×10^9^/L	9.00(6.18–13.4)	7.10(4.80–10.1)	0.002	6.20(4.40–8.80)	8.50(6.40–12.6)	9.40(6.88–12.5)	<0.001
Uric acid, μmol/L	379.8(274.0–499.4)	311.6(232.0–405.1)	0.005	319.3(240.8–409.5)	311.9(215.2–422.7)	445.5(282.4–609.6)	0.034
Urea nitrogen, mmol/L	7.82(5.68–11.5)	6.35(4.83–9.01)	0.005	5.91(4.80–8.51)	6.98(5.11–9.57)	10.0(6.35–22.3)	0.001
Triglycerides, mmol/L	1.27(1.00–1.77)	0.96(0.77–1.27)	<0.001	0.97(0.79–1.32)	1.12(0.76–1.38)	1.13(0.74–1.81)	0.535
Procalcitonin, ng/ml	0.25(0.05–2.28)	0.21(0.05–1.27)	0.474	0.11(0.05–0.65)	0.38(0.06–2.88)	0.65(0.18–5.06)	<0.001
CRP, mg/L	41.3(10.6–104.3)	44.4(12.2–99.4)	0.982	34.3(8.97–85.0)	62.7(15.9–110.2)	64.4(7.98–106.7)	0.024
D‐dimer, μg/ml	1.47(0.70–3.00)	1.70(0.85–3.00)	0.490	1.34(0.66–2.57)	2.06(1.18–3.12)	2.71(1.51–5.00)	<0.001

*Note*: Values were expressed as the median (interquartile range).

Abbreviation: CRP, C‐reactive protein.

### 3.3 Clinical outcomes

**FIGURE 2:**
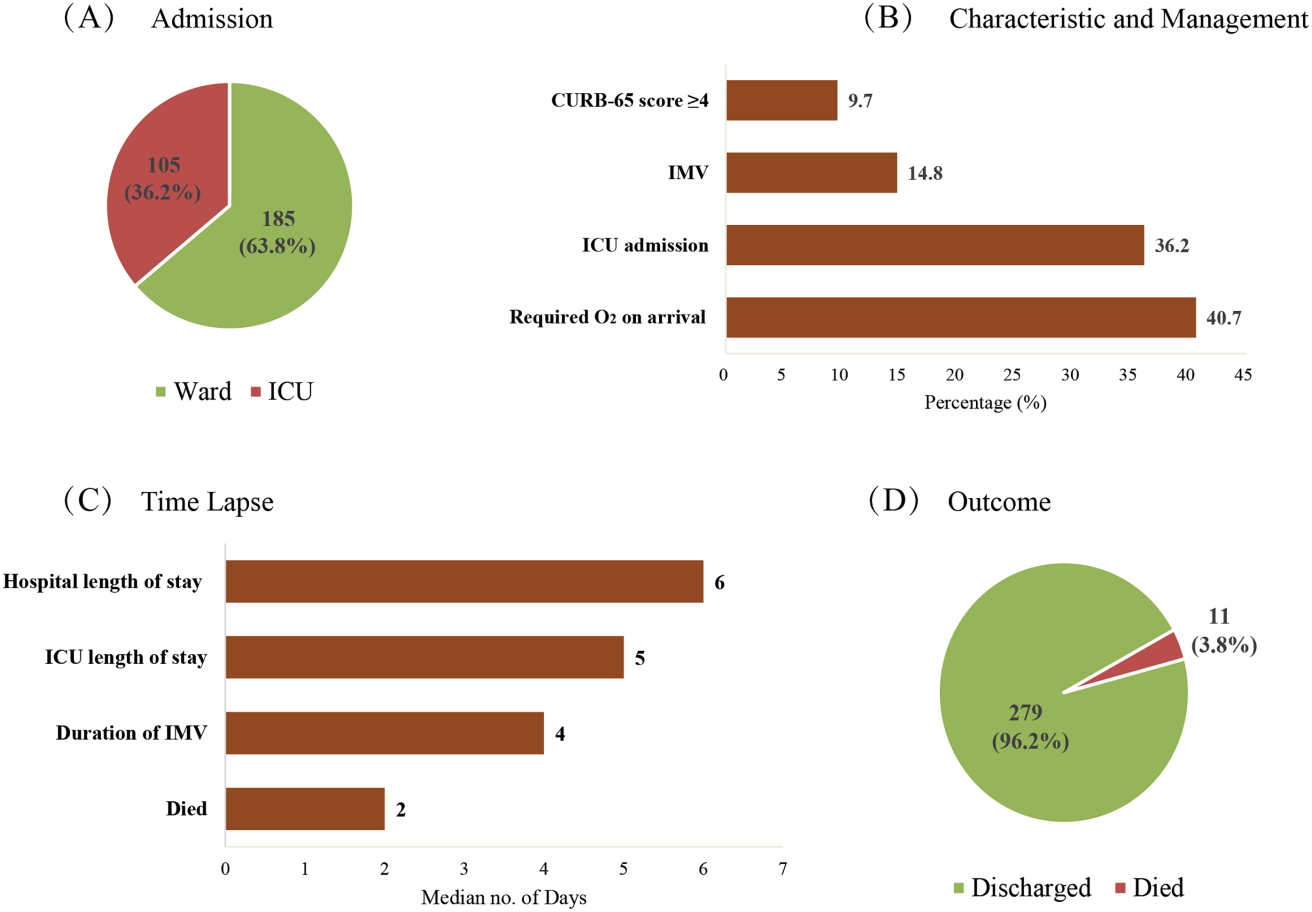
Management and outcomes of all patients (*n* = 290). (A) Percentage (%) of patients admitted to ward and intensive care unit (ICU); (B) percentage (%) of patients who received (X) intervention; (C) median number of days from admission for patients; (D) final outcome of patients

Of the 290 patients hospitalized with CAP, 185 (63.8%) were admitted to the general ward, and 105 (36.2%) were admitted to ICU, respectively. During the admission, 28 (9.7%) patients had a CURB‐65 score ≥ 4, 118 (40.7%) patients required O_2_ on arrival, and 43 (14.8%) patients needed IMV. The average length of stays from admission to discharge was 6 days. The average length of ICU stays in the hospital was 5 days, and the average duration of IMV was 4 days, respectively. Of these patients, there were 11 (3.8%) reported death in the patients with CAP, and the average time from admission to death was 2 days. These data are described in detail in Figure 2.

### 3.4 Risk factors for clinical outcomes

As shown in Table 3, after adjusting for age and sex, logistic regression analysis was utilized to identify the risk factors that might be associated with clinical outcomes in elderly patients with CAP. From our analysis, several risk factors related to 30‐day mortality or the incidence of ICU admission were chronic kidney disease, sepsis, and urea nitrogen > 8.2 mmol/L (*P* < 0.01). Meanwhile, we also found various risk factors associated with required IMV including chronic kidney disease, chronic liver disease, sepsis, neutrophil count > 6.3 × 10^9^/L, urea nitrogen > 8.2 mmol/L, and procalcitonin > 0.5 ng/ml (*P* < 0.01).

**TABLE 3:** Factors for outcomes of interest using the multivariate logistic regression analysis

Risk factor	Outcomes					
	30‐day mortality		ICU admission		Invasive mechanical ventilation	
	OR (95% CI)	*P* value	OR (95% CI)	*P* value	OR (95% CI)	*P* value
Pre‐existing comorbidities						
Chronic kidney disease	14.8 (3.0–72.6)	0.001	3.85 (2.15–6.89)	<0.001	6.21 (3.09–12.5)	<0.001
Chronic liver disease	4.37 (0.80–23.7)	0.088	2.76 (1.11–6.91)	0.030	7.78 (2.94–20.6)	<0.001
Sepsis	6.82 (1.71–27.3)	0.007	4.30 (1.58–11.7)	0.004	8.58 (3.23–22.8)	<0.001
Laboratory parameters						
Neutrophil count, >6.3 × 10^9^/L	0.93 (0.26–3.37)	0.911	1.41 (0.84–2.34)	0.191	3.06 (1.35–6.93)	0.007
Urea nitrogen, >8.2 mmol/L	20.5 (2.55–164.5)	0.004	2.39 (1.43–3.97)	0.001	3.78 (1.92–7.42)	<0.001
Procalcitonin, >0.5 ng/ml	14.5 (1.76–119.6)	0.013	1.65 (0.99–2.74)	0.055	3.71 (1.78–7.72)	<0.001

Abbreviations: CI, confidence interval; ICU, intensive care unit; OR, odds ratio.

### 3.5 Associations between diabetes, admission glucose level, and clinical outcomes

Among all patients, as is presented in Table 4, after adjusting for age and sex, multivariable logistic regression analysis revealed that admission glucose level > 11.1 mmol/L was significantly associated with ICU admission, IMV, and 30‐day mortality both in non‐diabetic and diabetic patients (Table 4). In addition, the adjusted odds ratios (ORs) for the association between admission glucose level (>6.1 mmol/L) and IMV in the general population were 2.87 (95% CI: 1.42–5.81, *P* = 0.003) and 3.20 (95% CI: 1.50–6.82, *P* = 0.003) in the non‐diabetic patients. Furthermore, patients with admission glucose level > 11.1 mmol/L had a significantly higher ICU admission, IMV, and 30‐day mortality than in the patients with admission glucose level ≤ 11.1 mmol/L, regardless of whether there is a known history of diabetes.

**TABLE 4:** Association between blood glucose levels and severe outcome in patients with and without diabetes mellitus

Variables	Adjusted OR (95% CI)	*P* value
ICU admission		
Blood glucose >6.1 mmol/L	0.67 (0.41–1.09)	0.110
Blood glucose >11.1 mmol/L	3.02 (1.31–6.99)	0.010
Nondiabetes patients		
Blood glucose >6.1 mmol/L	0.74 (0.42–1.30)	0.292
Blood glucose >11.1 mmol/L	6.78 (1.34–34.2)	0.020
Diabetes patients		
Blood glucose >6.1 mmol/L	0.82 (0.19–3.63)	0.821
Blood glucose >11.1 mmol/L	4.34 (1.20–15.7)	0.025
Invasive mechanical ventilation		
Blood glucose >6.1 mmol/L	2.87 (1.42–5.81)	0.003
Blood glucose >11.1 mmol/L	5.52 (2.20–13.9)	<0.001
Nondiabetes patients		
Blood glucose >6.1 mmol/L	3.20 (1.50–6.82)	0.003
Blood glucose >11.1 mmol/L	10.1 (2.39–42.5)	0.002
Diabetes patients		
Blood glucose >6.1 mmol/L	‐	‐
Blood glucose >11.1 mmol/L	8.40 (1.50–46.9)	0.015
In‐hospital mortality		
Blood glucose >6.1 mmol/L	3.15 (0.79–12.5)	0.103
Blood glucose >11.1 mmol/L	18.5 (4.21–80. 9)	<0.001
Nondiabetes patients		
Blood glucose >6.1 mmol/L	2.56 (0.59–11.1)	0.212
Blood glucose >11.1 mmol/L	14.4 (2.12–98.4)	0.006
Diabetes patients		
Blood glucose >6.1 mmol/L	‐	
Blood glucose >11.1 mmol/L	‐	

The ORs of blood glucose >6.1 mmol/L were with reference to patients with glucose ≤6.1 mmol/L; the ORs of blood glucose >11.1 mmol/L were with reference to patients with glucose ≤11.1 mmol/L.

Abbreviations: CI, confidence interval; ICU, intensive care unit; OR, odds ratio.

**FIGURE 3:**
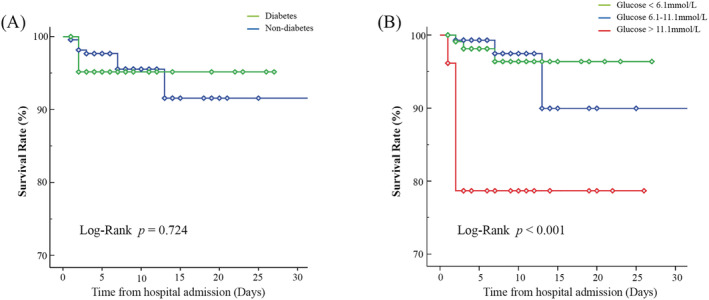
Kaplan–Meier curves of survival rate for patients. (A) Kaplan–Meier curves for community‐acquired pneumonia (CAP) patients with or without diabetes. (B) Kaplan–Meier curves for CAP patients with different admission blood glucose level

The relationship between admission glucose level, diabetic status, and 30‐day mortality was also evaluated using a Kaplan–Meier analysis, as shown in Figure 3. No statistically significant difference was found in the 30‐day mortality between the patients with and without a history of diabetes (*P* = 0.724). However, higher admission glucose level was correlated statistically significantly with 30‐day mortality in elderly patients with CAP (*P* < 0.001).

## 4 DISCUSSION

The incidence of CAP is increasing and is deemed to be the significant cause of infection‐related mortality worldwide. This study investigated the association between admission blood glucose level and clinical outcomes, including 30‐day mortality, ICU admission, and IMV, in elderly CAP patients with or without diabetes. Our results identified elevated blood glucose level on admission as significant predictors in all outcomes of interest (30‐day mortality, ICU admission, and IMV). Further, Kaplan–Meier analysis revealed that the 30‐day mortality after admission for CAP was related to higher glucose level determined on admission, irrespective of diabetes status at presentation. Therefore, admission blood glucose level might serve as an independent highly significant parameter for the prognosis of elderly CAP patients and particular attention of clinicians should be paid to these patients.

A large population‐based study reported that among patients hospitalized with pneumonia, of which 21% were admitted to ICU, 6% required IMV, and 2% died.^2^ Our study results found that (36.2%) patients were admitted to the ICU, 14.8% patients required IMV, and 3.8% died, which is higher than that in the aforementioned report. The disease severity of patients admitted with CAP is highly influenced by age and comorbidities.^13^ The elderly may be accompanied by a variety of basic diseases, such as cardiovascular and cerebrovascular disease, hypertension, diabetes, and renal that failure may exacerbate the degree of the disease and seriously affect the prognosis of the CAP patient.^14^, ^15^ Similar to many studies throughout the world, we found the complications including hypertension and cardiovascular disease were more commonly observed in patients with diabetes. It was noted that chronic kidney disease, chronic liver disease, and sepsis may contribute to the adverse outcomes of elderly CAP patients in this population; this is consistent with previous literature reports.^16^

Patients with infectious diseases frequently present with abnormal blood glucose level, especially in influenza and pneumonia.^10^, ^11^ Increasing evidence suggests that hyperglycemia is associated with higher mortality and adverse short‐term outcomes across a spectrum of critical illnesses.^17^, ^18^, ^19^ Patients with elevated glucose level are likely to be accompanied by a huge increase of inflammatory mediators and the impaired activation of neutrophils and phagocytosis, which ultimately give rise to exacerbating infection status.^20^, ^21^ Besides, stress hyperglycemia is further aggravated by pancreatic islet cell injury or hepatogenic insulin resistance.^22^ It is likely then, these conditions will become the major risk factor for critical illnesses and their poor prognosis. However, despite numerous studies that have documented these associations, shreds of evidences on the relationship between hyperglycemia on admission and the prognosis in patients with acute respiratory illness remain elusive, especially in patients with the presence or absence of diabetes.

Importantly, there is also debate on the relationship between admission glucose fluctuation and CAP. For example, one earlier study found that admission hyperglycemia was a strong predictor of 30‐day mortality in both pneumonia patients with and without diabetes.^23^ Similar to the above study, our results suggested that elevated admission glucose level was significantly associated with a higher risk of ICU admission, IMV, and 30‐day hospital mortality in elderly patients with CAP, regardless of the presence of diabetes. Further Kaplan–Meier survival analysis also demonstrated that elderly CAP patients with admission glucose >11.1 mmol/L had an increased risk of death. However, Jensen et al. reported that only in patients without diabetes, an elevated admission blood glucose was associated with risk for ICU admittance and a trend towards higher in‐hospital mortality.^24^ Lepper et al. also reported that an increased serum glucose level at admission was an independent predictor of death at 28 and 90 days in patients with CAP without pre‐existing diabetes.^25^ A recent study reported by Cheng et al. indicated that hyperglycemia is one of the factors for in‐hospital mortality among patients with diabetes mellitus and concomitant CAP.^26^ Conversely, Bhattacharya et al. found that admission glucose level was not associated with adverse outcomes within 30 days in older patients with CAP.^27^ As the above studies were limited to the specific geographical area or single healthcare system, therefore, it is difficult to reach a unanimous conclusion on the research results. The reasons for these discrepancies are presumed to be multifactorial, such as the study population, absence of standard definitions, lack of control cohorts and selection or information biases makes it difficult to consistently determine.

The following limitations of the study have to be mentioned. Firstly, our study is a retrospective study with relatively small sample at a single center; therefore, our findings may not be broadly applicable. In addition, we measured only blood glucose at admission, not dynamic blood glucose during hospitalization non‐dynamic observation of blood glucose during hospitalization may be another reason for this result. Further clinical research of larger samples is still needed to confirm our results.

In conclusion, we found that admission blood glucose is correlated with in‐hospital mortality, ICU admission, and IMV of CAP in elderly patients with and without diabetes. In addition, admission glucose > 11.1 mmol/L was a significant risk factor for death in elderly patients with CAP. Our study suggests that admission blood glucose may be used as an important and useful clinical tool for early and effective risk assessment of CAP in elderly patients, and further studies should be conducted to evaluate this possibility.

## CONFLICT OF INTEREST

The authors declare that they have no competing interests. No conflict of interest exits in the submission of this manuscript, and all the authors listed have approved the manuscript that is enclosed. The manuscript has been approved by all authors for publication.

## ETHICS STATEMENT

All procedures complied with the ethical guidelines outlined in the 1975 Helsinki Declaration and approved by the Ethics Committee of Meizhou People's Hospital (Huangtang Hospital) (number: 2020‐C‐118). Patients' consent was waived due to the retrospective nature and observational study design.

## AUTHOR CONTRIBUTIONS

Author WZ contributed to study design and interpretation of results and revised the final manuscript for publication. XH and WZ contributed to the analysis of results and wrote the manuscript. WL and MC supervised patients' recruitment and collected the clinical data. All authors read and approved the final manuscript.

## Supporting information


**Table S1** Patients' demographic and clinical characteristics admission to the hospital.
**Table S2** Comparison of laboratory parameters among sub‐groups at admission

## Data Availability

The datasets generated and/or analyzed during the current study are not publicly available due to the fact that individual privacy could be compromised but are available from the corresponding author on reasonable request.
