# Supplementary material for: Association of admission blood glucose level and clinical outcomes in elderly community‐acquired pneumonia patients with or without diabetes
Source: Clin Respir J. 2022 Jul 24;16(8):562–71. doi: 10.1111/crj.13526 (PMC9376138; doi:10.1111/crj.13526)
Supplement: Supplementary file 1 — Table S1 Patients' demographic and clinical characteristics admission to the hospital. Table S2 Comparison of laboratory parameters among sub‐groups at admission [file CRJ-16-562-s001.docx]

**TABLE S1** Patients’ demographic and clinical characteristics admission to the hospital.

|  | Diabetes  (n = 64) | Non-diabetes  (n = 226) | *P* value |  | Blood glucose <6.1mmol/L  (n = 147) | Blood glucose 6.1-11.1mmol/L  (n = 117) | Blood glucose＞11.1mmol/L  (n = 26) | *P* value |
| --- | --- | --- | --- | --- | --- | --- | --- | --- |
| **Pre-existing comorbidities, n (%)** |  |  |  |  |  |  |  |  |
| Valvular heart disease | 10 (15.6) | 40 (17.7) | 0.698 |  | 30 (20.4) | 17 (14.5) | 3 (11.5) | 0.328 |
| Arrhythmia | 6 (9.4) | 30 (13.3) | 0.404 |  | 14 (9.5) | 17 (14.5) | 5 (19.2) | 0.256 |
| Cerebrovascular disease | 19 (29.7) | 52 (23.0) | 0.273 |  | 36 (24.5) | 24 (20.5) | 11 (42.3) | 0.065 |
| Sepsis | 3 (4.7) | 17 (7.5) | 0.610 |  | 8 (5.4) | 8 (6.8) | 4 (23.4) | 0.182 |
| Malignancy | 8 (12.5) | 25 (11.1) | 0.749 |  | 22(15.0) | 8 (6.8) | 3 (11.5) | 0.118 |
| **Symptoms at presentation, n (%)** |  |  |  |  |  |  |  |  |
| Fever (≥38℃) | 15 (23.4) | 33 (14.6) | 0.093 |  | 20 (13.6) | 22 (18.8) | 6 (23.1) | 0.340 |
| Cough | 55 (85.9) | 198 (87.6) | 0.723 |  | 134 (91.2) | 98 (83.8) | 21 (80.8) | 0.118 |
| Sputum production | 6 (9.4) | 29 (12.8) | 0.454 |  | 16 (10.9) | 13 (11.1) | 6 (23.1) | 0.196 |

*Values are expressed as the number (percentage).*

**TABLE S2** Comparison of laboratory parameters among sub-groups at admission

|  | Diabetes  (n = 64) | Non-diabetes  (n = 226) | *P* value | Blood glucose  < 6.1mmol/L  (n = 147) | Blood glucose  6.1-11.1mmol/L  (n = 117) | Blood glucose  ＞11.1mmol/L  (n = 26) | *P* value |
| --- | --- | --- | --- | --- | --- | --- | --- |
| Lymphocyte count, ×10⁹/L | 1.30(0.80-1.90) | 1.10(0.70-1.60) | 0.030 | 1.20(0.80-1.76) | 1.10(0.60-1.60) | 0.85(0.60-1.75) | 0.131 |
| Monocyte count, ×10⁹/L | 0.70(0.50-1.08) | 0.60(0.40-0.90) | 0.072 | 0.60(0.43-0.90) | 0.70(0.40-1.00) | 0.70(0.40-0.93) | 0.691 |
| Platelet count, ×10⁹/L | 226.5(159.0-298.0) | 207.0(155.0-270.3) | 0.190 | 219.0(155.0-273.0) | 207.0(161.0-259.0) | 204.0(130.5-316.0) | 0.646 |
| Creatinine, μmol/L | 104.9(82.5-138.4) | 92.0(72.8-121.1) | 0.021 | 92.0(75.8-116.7) | 96.90(72.6-126.8) | 113.15(78.6-187.4) | 0.121 |
| Total cholesterol, mmol/L | 3.74(3.03-4.85) | 3.85(3.04-4.74) | 0.879 | 3.92(3.16-4.92) | 3.75(2.95-4.63) | 3.17(2.07-3.81) | 0.054 |
| HDL-cholesterol, mmol/L | 1.12(0.91-1.39) | 1.13(0.94-1.36) | 0.980 | 1.16(0.95-1.38) | 1.14(0.93-1.35) | 1.01(0.64-1.32) | 0.206 |
| LDL-cholesterol, mmol/L | 2.19(1.64-3.01) | 2.06(1.68-2.79) | 0.265 | 2.10(1.76-2.87) | 2.12(1.65-2.79) | 1.87(0.92-2.26) | 0.161 |
| Apo-A1, g/L | 1.00(0.76-1.21) | 0.93(0.72-1.13) | 0.364 | 0.96(0.77-1.22) | 0.92(0.66-1.12) | 0.93(0.63-1.06) | 0.198 |
| ApoB, g/L | 0.78(0.56-1.02) | 0.71(0.56-0.92) | 0.314 | 0.73(0.55-0.94) | 0.75(0.59-0.93) | 0.68(0.52-0.79) | 0.212 |
| Albumin, g/L | 34.8(31.2-37.4) | 33.1(28.9-36.6) | 0.074 | 33.5(29.6-36.7) | 33.6(29.2-37.8) | 32.5(25.2-36.3) | 0.456 |

Values were expressed as the median (interquartile range).

Abbreviations: Apo-A1, Apolipoprotein A1; ApoB, Apolipoprotein B; HDL, High-density lipoprotein; LDL, Low-density lipoprotein.
